# Cranial MRI Findings in Rheumatoid Arthritis Patients Treated with Rituximab: Prevalence, Patterns, and Clinical Correlates

**DOI:** 10.3390/jcm15197490

**Published:** 2026-09-26

**Authors:** Belkıs Nihan Coşkun, Rıfat Özpar, Nihal Lermi, Gizem Güllü, Selime Ermurat, Zeynep Yılmaz Bozkurt, Burcu Yağız, Özlem Taşkapılıoğlu, Yavuz Pehlivan, Ediz Dalkılıç

**Affiliations:** 1 Division of Rheumatology, Department of Internal Medicine, Faculty of Medicine, Bursa Uludağ University, 16059 Bursa, Türkiyeburcuyilmaz_84@hotmail.com (B.Y.);; 2 Department of Radiology, Faculty of Medicine, Bursa Uludağ University, 16059 Bursa, Türkiye; 3 Department of Neurology, Bursa Yüksek İhtisas Training and Research Hospital, 16310 Bursa, Türkiye; 4 Division of Rheumatology, Department of Internal Medicine, Bursa Yüksek İhtisas Training and Research Hospital, 16310 Bursa, Türkiye; 5 Department of Neurology, Faculty of Medicine, Bursa Uludağ University, 16059 Bursa, Türkiye

**Keywords:** rheumatoid arthritis, rituximab, magnetic resonance imaging, central nervous system, white matter, comorbidity

## Abstract

**Background/Objectives:** Neurological manifestations in rheumatoid arthritis (RA) are common, yet the clinical relevance of central nervous system (CNS) abnormalities detected on neuroimaging remains uncertain. We aimed to determine the prevalence and patterns of cranial magnetic resonance imaging (MRI) findings in RA patients treated with rituximab (RTX) and to assess whether these abnormalities are associated with disease-specific mechanisms or with age- and comorbidity-related factors. **Methods:** In this retrospective observational study, 84 RA patients who received RTX and had available pre-treatment cranial MRI underwent systematic evaluation. Imaging findings were classified by an experienced neuroradiologist blinded to clinical data and dichotomized as normal or abnormal, and white matter hyperintensities were graded semiquantitatively using a Fazekas-type scale. Factors independently associated with abnormal MRI were assessed by multivariable logistic regression. **Results:** Cranial MRI abnormalities were identified in 60 patients (71.5%), predominantly as nonspecific white matter hyperintensities (48 patients, 57.1%). In multivariable analysis, older age (adjusted odds ratio [aOR] 1.16 per year, 95% CI 1.08–1.25) and the presence of comorbidities (aOR 5.36, 95% CI 1.49–19.23) were independently associated with abnormal imaging (*p* < 0.001 and *p* = 0.010, respectively), whereas no association was found with disease duration, seropositivity, or extra-articular involvement. Imaging findings remained stable over time in the majority of the 39 patients (46.4%) who underwent follow-up imaging. **Conclusions:** Cranial MRI abnormalities are highly prevalent in RA and were associated primarily with age and comorbidity burden rather than with RA-specific inflammation; however, in the absence of a control group, we cannot determine whether their prevalence exceeds that of a comparable non-RA population. No treatment-related demyelinating disease or progressive multifocal leukoencephalopathy was observed, but the study was not designed to assess drug safety, and these findings should be interpreted as the absence of an observed imaging signal rather than as evidence of the neurological safety of rituximab. Cranial neuroimaging in RA should therefore be interpreted cautiously and in clinical context.

## 1. Introduction

Rheumatoid arthritis (RA) is a chronic, systemic autoimmune disease characterized by symmetrical polyarthritis, persistent synovitis, and progressive joint destruction. Beyond the joints, RA gives rise to a broad spectrum of extra-articular manifestations affecting the skin, lungs, eyes, cardiovascular system, and nervous system, reflecting the systemic, immune-mediated nature of the disease and contributing to its associated morbidity and mortality [1,2]. Neurological involvement, in particular, constitutes a clinically important but heterogeneous domain that spans both the peripheral and the central nervous systems (CNS).

Peripheral neuropathy is the most frequently reported neurological complication of RA and is often subclinical, being detected on electrophysiological testing in a substantial proportion of patients even in the absence of overt symptoms; reported prevalences range widely, from approximately one-third to three-quarters of patients, depending on the diagnostic method and the population studied [3,4]. Axonal sensory and sensorimotor neuropathies predominate, together with entrapment neuropathies such as carpal tunnel syndrome, and these are attributed to vasculitis of the vasa nervorum, chronic systemic inflammation, and mechanical factors [5].

In contrast to peripheral involvement, CNS manifestations of RA are less well defined and are frequently under-recognized in routine clinical practice. The reported spectrum includes cervical spine pathology, such as atlantoaxial subluxation and pannus formation [6]; cognitive impairment, which affects a considerable proportion of patients and exceeds the rates observed in matched controls [7]; central sensitization and neuropathic pain [8,9]; and rare but serious inflammatory conditions such as rheumatoid meningitis and cerebral vasculitis [10,11,12]. In parallel, neuroimaging studies have increasingly demonstrated structural brain changes in RA, including white matter abnormalities, cerebral small-vessel lesions, and brain atrophy [13].

Among these, white matter hyperintensities (WMH) on cranial MRI are the most commonly encountered abnormality, yet their clinical significance and pathophysiology remain controversial. WMH may reflect disease-specific injury related to chronic systemic inflammation and endothelial dysfunction [5], or they may be largely attributable to age-related and comorbidity-associated small-vessel disease that is also prevalent in the general population. Reported associations between CNS imaging findings and RA-specific characteristics—disease duration, seropositivity, and disease activity—have been inconsistent across studies, and relatively few studies have evaluated these relationships systematically within a well-defined treatment cohort.

Rituximab (RTX), a chimeric monoclonal antibody directed against CD20-positive B cells, is an established therapy for RA, particularly in patients with an inadequate response to conventional synthetic disease-modifying antirheumatic drugs (csDMARDs) or tumor necrosis factor inhibitors. Although rare neurological adverse events—most notably progressive multifocal leukoencephalopathy (PML), a demyelinating brain infection caused by the JC virus—have been reported in RA patients receiving RTX [14,15], its overall neurological safety profile is considered favorable. Nevertheless, data on baseline cranial MRI findings in RA patients before and during RTX therapy remain scarce, and it is unclear whether pre-existing CNS abnormalities in such patients are disease-related or reflect background vascular risk.

A clearer understanding of the prevalence, patterns, and determinants of cranial MRI abnormalities in RA is therefore needed—both to guide the interpretation of incidental neuroimaging findings and to establish a baseline against which potential treatment-related CNS changes may be judged. Against this background, the present study had three objectives: (i) to determine the prevalence and patterns of cranial MRI abnormalities in a well-characterized cohort of RA patients treated with RTX; (ii) to examine whether these abnormalities are associated with RA-specific parameters (disease duration, seropositivity, and extra-articular involvement) or with age and comorbidity burden; and (iii) to assess the longitudinal stability of imaging findings during follow-up. We hypothesized that cranial MRI abnormalities would be common but driven predominantly by age- and comorbidity-related mechanisms rather than by RA-specific inflammation.

## 2. Materials and Methods

### 2.1. Study Design and Population

This was a single-center, retrospective, observational cohort study conducted at the Division of Rheumatology, Bursa Uludağ University Faculty of Medicine, a tertiary referral center. In Türkiye, between 2006 and 2016, neurological clearance documented on the drug-safety form was required before rituximab could be initiated, and cranial MRI was routinely requested for this purpose. Eligible patients were identified retrospectively as those whose pre-treatment cranial MRI had been requested before the date of ethics committee approval (14 April 2015; Approval No: 2015-9/11); owing to the long waiting times for MRI appointments in our setting, some of these requested examinations were performed subsequently, so that the actual imaging dates ranged from 2006 to 2016. The retrospective collection and analysis of the data were carried out only after ethics approval had been obtained (approved study start date, 1 May 2015). Consecutive patients were considered eligible if they (i) were adults (≥18 years) with a diagnosis of RA fulfilling the 2010 American College of Rheumatology/European League Against Rheumatism (ACR/EULAR) classification criteria; (ii) had received RTX therapy; and (iii) had an evaluable pre-treatment cranial MRI examination available in the institutional imaging archive. Patients with incomplete clinical records or without an interpretable pre-treatment cranial MRI were excluded. No specific additional exclusion criteria were applied. These cranial MRI examinations were thus obtained systematically for pre-treatment neurological clearance rather than in response to neurological symptoms, which minimizes selection bias toward symptomatic patients. In total, 84 patients met these criteria and formed the study cohort.

Patients were identified from the rheumatology outpatient clinic database, and their radiological images were reviewed retrospectively using the institutional Picture Archiving and Communication System (PACS; Centricity RIS 4.2 Plus PACS system, General Electric, Chicago, IL, USA). Clinical and demographic data—including age, sex, disease duration, serological status (rheumatoid factor [RF] and anti-cyclic citrullinated peptide [anti-CCP] antibodies), comorbidities, extra-articular manifestations, and RTX treatment history—were extracted from the electronic medical records. RTX was administered as two 1000 mg intravenous infusions given two weeks apart, with treatment courses repeated at approximately 6-month intervals according to clinical response.

### 2.2. MRI Acquisition and Evaluation

Cranial MRI examinations were performed as part of routine clinical care before the initiation of RTX. All MRI examinations were performed using a 3-T MRI system (Achieva TX, Philips Healthcare, Best, The Netherlands). The imaging protocol included pre- and post-contrast T1-weighted spin-echo (SE) imaging (TR/TE, 500/7 ms; voxel size, 0.7 × 1 × 5 mm), T2-weighted turbo spin-echo (TSE) imaging (TR/TE, 3500/90 ms; voxel size, 0.7 × 1 × 5 mm), fluid-attenuated inversion recovery (FLAIR) TSE imaging (TR/TE/TI, 11,000/125/2800 ms; voxel size, 0.7 × 1 × 5 mm), and diffusion-weighted imaging (DWI) (TR/TE, 3789/88.3 ms; voxel size, 1.6 × 2 × 5 mm). For contrast-enhanced imaging, gadoterate meglumine was administered intravenously at a dose of 0.2 mL/kg and an injection rate of 2.5 mL/s. All images were retrieved and reviewed on the PACS workstation by an experienced neuroradiologist (with 10 years of experience in neuroradiology) who was blinded to the patients’ clinical and laboratory data.

Imaging findings were categorized according to lesion characteristics and anatomical localization into the following predefined groups: (1) normal findings; (2) nonspecific white matter hyperintensities (WMH); (3) demyelinating-like lesions; (4) ischemic lesions; (5) vasculitic patterns; (6) mass or nodular lesions; and (7) infectious lesions. When multiple abnormalities coexisted, the case was classified according to the most clinically significant finding. For patients with more than one cranial MRI examination available, follow-up images were systematically compared with the baseline study to assess the temporal evolution of findings. Overall, 39 of the 84 patients (46.4%) had at least one follow-up cranial MRI examination in addition to the baseline study. Radiological definitions were applied as follows: white matter hyperintensities (WMH) were defined as focal or confluent T2/FLAIR hyperintensities within the white matter, subclassified by location as periventricular, deep, or subcortical; cortical atrophy as sulcal widening and volume loss disproportionate to age; chronic lacunar (ischemic) lesions as small (<15 mm) cavities with cerebrospinal-fluid signal; demyelinating-like lesions as ovoid periventricular lesions oriented perpendicular to the ventricles; vasculitic patterns as multifocal cortical/subcortical infarcts or vessel-wall abnormalities; and mass/nodular or infectious lesions according to standard criteria. To provide a semiquantitative measure of lesion burden, white matter hyperintensities were graded using a Fazekas-type visual rating scale, in which periventricular and deep WMH were each graded from 0 to 3; subcortical WMH and cortical atrophy were graded from 0 to 3 in an analogous manner. All coexisting findings were recorded, not only the most clinically significant one. Repeat cranial MRI, when performed, was generally obtained at an interval of approximately 6–7 months, corresponding to the rituximab retreatment cycle.

### 2.3. Clinical Variables and Definitions

Comorbidities were defined as the presence of one or more chronic conditions—including hypertension, diabetes mellitus, hyperlipidemia, coronary artery disease, cerebrovascular disease, chronic renal failure, or other clinically documented systemic diseases—as recorded in the medical records. Seropositivity was defined as a positive RF and/or anti-CCP result according to the local laboratory reference ranges. Extra-articular involvement was defined on the basis of physician documentation in the medical records. For the statistical analyses, cranial MRI findings were dichotomized as normal or abnormal; in a secondary analysis, patients were additionally grouped according to the presence or absence of white matter disease (WMD), the most frequent abnormal subtype. In addition to this composite variable, the individual comorbid conditions (hypertension, diabetes mellitus, hyperlipidemia, coronary artery disease, cerebrovascular disease, and chronic renal failure) were examined separately with respect to MRI findings.

### 2.4. Statistical Analysis

Statistical analyses were performed using IBM SPSS Statistics (version 23.0; IBM Corp., Armonk, NY, USA). The normality of continuous variables was assessed with the Kolmogorov–Smirnov test. Continuous variables were expressed as mean ± standard deviation or as median (minimum–maximum), as appropriate, and categorical variables as frequencies and percentages. Between-group comparisons (normal vs. abnormal MRI, and WMD present vs. absent) were performed using the independent-samples *t*-test or the Mann–Whitney U test for continuous variables, and the chi-square test or Fisher’s exact test for categorical variables, as appropriate. Receiver operating characteristic (ROC) curve analysis was used to evaluate the discriminative performance of age and of comorbidity burden for the presence of abnormal cranial MRI findings, and the area under the curve (AUC) was calculated to quantify discriminative ability. The optimal age threshold for predicting abnormal cranial MRI findings was determined using the Youden index. A two-sided *p*-value < 0.05 was considered statistically significant. To identify factors independently associated with abnormal cranial MRI findings, a multivariable binary logistic regression model was fitted with abnormal MRI as the dependent variable and age and comorbidity as covariates; results are reported as adjusted odds ratios (aOR) with 95% confidence intervals (CI). Because the number of outcome events was limited, the model was deliberately kept parsimonious, and a sensitivity analysis additionally adjusting for sex and disease duration was performed. During the preparation of this manuscript, the authors used a generative artificial-intelligence tool (Claude; Anthropic PBC) to assist with English-language editing, formatting of the manuscript to the journal template, and the preparation and independent verification of the descriptive and statistical analyses; all outputs were checked against the authors’ own analyses, and the authors reviewed and take full responsibility for the content and conclusions.

### 2.5. Ethical Approval

The study was conducted in accordance with the Declaration of Helsinki and approved by the Clinical Research Ethics Committee of Bursa Uludağ University Faculty of Medicine (Approval No: 2015-9/11; Date: 14 April 2015). Owing to the retrospective design, the requirement for informed consent was waived. In accordance with the retrospective design, all data collection and analysis were performed only after this approval had been obtained.

## 3. Results

### 3.1. Patient Characteristics

A total of 84 patients with RA were included. The demographic and clinical characteristics of the study population are summarized in Table 1. Patients were subsequently stratified into two groups according to cranial MRI findings (normal vs. abnormal), and the comparison of these groups is presented in Table 2.

### 3.2. Prevalence of Cranial MRI Abnormalities

Cranial MRI abnormalities were detected in 60 patients (71.5%), whereas 24 patients (28.5%) had normal findings. The predominant abnormality was nonspecific white matter hyperintensity (WMH), present in 48 patients (57.1%). On semiquantitative (Fazekas-type) grading, deep WMH were present in 41 patients (grade 1, 28 [33.3%]; grade 2, 12 [14.3%]; grade 3, 1 [1.2%]), periventricular WMH in 8 (grade 1, 4 [4.8%]; grade 2, 4 [4.8%]), and subcortical WMH in 12 (grade 1, 7 [8.3%]; grade 2, 5 [6.0%]); the graded distribution is summarized in Table 3. Cortical atrophy was present in 17 patients (20.2%; predominantly grade 1; grade data unavailable in one patient) and chronic lacunar (ischemic) lesions in 10 (11.9%). In addition, eight patients (9.5%) each showed an individually uncommon finding: a left frontal contusion sequela, a left temporal cortical contusion sequela, normal-pressure hydrocephalus, prominent perivascular spaces, a brainstem hyperintensity, cerebellar atrophy, cerebral venous sinus thrombosis, and a left frontal metastasis secondary to breast cancer. With the exception of this single metastatic lesion, no mass, demyelinating, vasculitic, or infectious lesions were identified. Because individual patients frequently exhibited more than one finding, these categories are not mutually exclusive.

### 3.3. Association Between MRI Findings and Clinical Variables

Patients with abnormal MRI findings were significantly older than those with normal MRI (*p* < 0.001). In addition, the presence of comorbidities was significantly higher in patients with abnormal MRI findings (*p* = 0.001). No statistically significant differences were observed between the groups in terms of sex, disease duration, RF positivity, anti-CCP positivity, or extra-articular involvement (*p* > 0.05 for all) (Table 2).

When the individual comorbid conditions were examined separately, each was numerically more frequent among patients with abnormal MRI than among those with normal MRI (hypertension 41.7% vs. 20.8%; diabetes mellitus 20.0% vs. 8.3%; hyperlipidemia 11.7% vs. 4.2%; coronary artery disease 10.0% vs. 0%; cerebrovascular disease 5.0% vs. 0%; chronic renal failure 1.7% vs. 0%), but none reached statistical significance individually (all *p* > 0.05), most likely reflecting the small number of patients with each condition. The composite comorbidity variable was therefore retained as the primary measure of vascular-risk burden.

### 3.4. Predictors of MRI Abnormalities

ROC curve analysis demonstrated that age had a strong predictive value for abnormal cranial MRI findings (AUC: 0.86), whereas the presence of comorbidities showed a moderate predictive value (AUC: 0.68) (Figure 1). Based on the Youden index, an age threshold of approximately 52 years provided the optimal balance between sensitivity (78.3%) and specificity (75.0%) for identifying abnormal cranial MRI findings. In multivariable logistic regression, both older age (aOR 1.16 per year, 95% CI 1.08–1.25; equivalently aOR 4.49 per 10-year increment, 95% CI 2.13–9.49; *p* < 0.001) and the presence of comorbidities (aOR 5.36, 95% CI 1.49–19.23; *p* = 0.010) remained independently associated with abnormal cranial MRI findings; these associations were essentially unchanged in a sensitivity analysis additionally adjusting for sex and disease duration (Table 4). It should be emphasized that ROC analysis reflects discrimination rather than causation or independent predictive value, and that the age threshold of approximately 52 years requires external validation in larger, unselected populations before any clinical application.

### 3.5. Follow-Up MRI Findings

Follow-up cranial MRI was available in 39 of the 84 patients (46.4%). Patients with and without follow-up imaging did not differ significantly in age (55.0 ± 11.3 vs. 54.4 ± 12.1 years, *p* = 0.82), sex (female, 92.3% vs. 82.2%), comorbidity burden (71.8% vs. 68.9%), or the proportion with an abnormal baseline MRI (74.4% vs. 68.9%), arguing against major indication bias; the repeat examinations were generally performed at an interval of approximately 6–7 months, corresponding to the rituximab retreatment cycle. Among the patients who underwent repeat imaging, cranial MRI findings remained stable in the majority. Notable progression or new pathological findings were observed in three patients: one developed multiple scattered cerebral infarcts without evidence of large-vessel occlusion; another demonstrated progression of cerebral atrophy and periventricular ischemic gliosis; and in a third, intracranial metastasis was detected in the context of a newly diagnosed malignancy. Apart from these cases, no new inflammatory or demyelinating CNS involvement was observed. Because follow-up was available in fewer than half of the cohort, this observation of stability applies only to the imaged subgroup and cannot be generalized to the entire study population. The interval between the baseline and follow-up examinations was approximately 6–7 months, corresponding to the rituximab retreatment cycle.

## 4. Discussion

In this study, we evaluated cranial MRI findings in RA patients before and during follow-up after RTX therapy, focusing on CNS involvement. We demonstrated that cranial MRI abnormalities are highly prevalent, being detected in more than two-thirds of the cohort, with nonspecific WMH representing the predominant pattern. Importantly, these abnormalities were primarily associated with age and comorbidity burden rather than RA-specific disease characteristics. These associations, however, were derived from a single-center cohort without a control group, and the wording throughout should be read as describing associations rather than establishing causation.

Neurological involvement in RA has long been recognized as a multifaceted and often underdiagnosed component of the disease. Previous studies have reported a broad spectrum of neurological manifestations, with peripheral neuropathy being the most common and frequently identified at subclinical stages [3,4]. Peripheral neurological injury in RA is thought to arise through several mechanisms, including vasculitis of the vasa nervorum, leading to vascular ischemia, axonal degeneration, and demyelination, and a substantial proportion of patients develop multiple mononeuritis or distal sensory and sensorimotor neuropathies [5]. In contrast, CNS involvement has been less consistently characterized, with reported manifestations including cognitive impairment [7], cervical spine pathology [6], and rare inflammatory conditions such as rheumatoid meningitis [10,11,12]. Neuroimaging studies have increasingly highlighted structural brain changes in RA patients, particularly white matter abnormalities and brain atrophy, supporting the concept of CNS involvement as part of the systemic disease process [13].

RA-related CNS manifestations such as aseptic meningitis, vasculitis, and cerebral rheumatoid nodules are, however, distinctly uncommon [10]. In our cohort of 84 patients with established, long-standing RA, none of these classic inflammatory CNS manifestations were observed. This is consistent with a large retrospective study of 519 hospitalized RA patients, in which the frequency of neurological involvement was as low as 0.39% [2]. Our findings therefore reinforce the notion that overt RA-specific CNS disease is rare, even in patients with advanced disease receiving biologic therapy.

The clinical significance and underlying mechanisms of incidental imaging findings nonetheless remain controversial. While some studies have suggested that CNS abnormalities may reflect chronic systemic inflammation and disease activity, others have reported inconsistent or weak associations with RA-specific parameters. Our findings support the latter perspective, as no significant relationship was observed between MRI abnormalities and disease duration, serological markers, or extra-articular involvement. The etiology and pathogenesis of extra-articular manifestations in RA are not yet fully elucidated; environmental factors such as smoking and dietary habits, male sex, and chronic systemic inflammation have all been proposed as contributing factors [1,2]. Although male sex has been associated with a higher burden of extra-articular involvement, we observed no difference between sexes in our cohort.

The predominance of WMH in our cohort is consistent with previous neuroimaging studies in RA. These lesions are commonly interpreted as markers of small-vessel disease and are frequently observed in aging populations and individuals with vascular risk factors. The strong association between WMH and age in our study, together with the contribution of comorbidities, suggests that microvascular mechanisms rather than direct inflammatory processes play a central role in the development of these abnormalities. Chronic systemic inflammation may still contribute indirectly by accelerating vascular aging and endothelial dysfunction, thereby increasing susceptibility to microvascular brain injury [5]. The strong predictive value of age (AUC: 0.86) aligns with reports of cerebral small-vessel lesions and brain atrophy in RA, which have been linked to vascular comorbidity burden and, indirectly, to chronic systemic inflammation [13]. Similarly, the moderate predictive value of comorbidities (AUC: 0.68) highlights the contribution of traditional cardiovascular and metabolic risk factors to CNS imaging abnormalities. Because our study lacked a control group, a contribution of RA-related mechanisms to these microvascular changes cannot be excluded, and the predominance of age and comorbidity should be interpreted as an association rather than proof that RA itself plays no role. The vascular comorbidities recorded in our cohort—particularly hypertension, diabetes mellitus, and hyperlipidemia—are well-established drivers of cerebral small-vessel disease and white matter hyperintensities, and provide a plausible non-inflammatory basis for the lesions observed.

Interestingly, RA-specific disease parameters were not associated with MRI abnormalities in our cohort. This finding contrasts with some studies suggesting associations between neurological involvement and disease activity, seropositivity, or disease duration [6,7]. Inconsistencies across studies may be explained by differences in patient populations, diagnostic modalities, and the inclusion of clinically overt versus subclinical manifestations. Notably, the interplay between neurological findings and disease activity is bidirectional: central sensitization and neuropathic pain, reported in roughly half of RA patients, can inflate composite disease-activity scores and lead to overestimation of inflammatory activity [8,9]. Our results suggest that, at least in the context of incidental MRI findings, non-inflammatory mechanisms such as microvascular damage may play a more prominent role.

Another important finding of our study is the relative stability of MRI abnormalities during follow-up. With the exception of three patients with distinct pathological processes (ischemic infarction, progressive gliosis, and intracranial metastasis), imaging findings remained unchanged over time, and no clear pattern of demyelinating disease or progressive inflammatory CNS involvement was observed. This observation further supports the notion that most MRI abnormalities in RA are non-inflammatory in nature.

The role of RTX in relation to CNS findings also warrants comment. RTX is a high-affinity chimeric monoclonal antibody specific for CD20 and was the first B-cell-targeted biologic disease-modifying antirheumatic drug approved for RA. Although rare neurological adverse events, particularly PML—a serious demyelinating brain infection caused by the JC virus—have been reported in RA patients receiving RTX [14,15], we observed no evidence of treatment-related demyelinating disease or progressive CNS pathology, and no case of PML was identified. This observation must, however, be interpreted with considerable caution. All patients received RTX, so no untreated or alternatively treated comparator group was available; follow-up imaging was obtained in fewer than half of the cohort; and the study was neither designed nor powered as a safety study. The absence of PML—an extremely rare event with a very low background incidence—in 84 patients is statistically expected and cannot establish neurological safety. These findings should therefore be regarded as the absence of an observed treatment-related imaging signal in this cohort rather than as evidence supporting the neurological safety of rituximab. Importantly, because the cranial MRI examinations were obtained before the initiation of rituximab (as pre-treatment neurological clearance), the baseline findings reflect the patients’ pre-treatment status and cannot, by design, be attributed to rituximab; only the follow-up imaging, available in a minority of patients, could reflect treatment-emergent change. Together with the retrospective design, this reinforces that our data should be read as the absence of an observed imaging signal rather than as evidence of a favorable neurological safety profile.

From a clinical perspective, our findings have several implications. First, the high prevalence of nonspecific MRI abnormalities highlights the need for cautious interpretation of neuroimaging in RA patients, particularly in older individuals with comorbidities; overinterpretation of such findings as disease-related CNS involvement may lead to unnecessary diagnostic procedures or inappropriate therapeutic decisions. Second, baseline MRI evaluation may be useful in selected patients, especially before initiating biologic therapy, to distinguish pre-existing abnormalities from potential treatment-related changes. However, the absence of significant progression at follow-up suggests that routine serial imaging may not be necessary in asymptomatic patients. In practical terms, these observations support an individualized, risk-stratified approach in which baseline cranial MRI is prioritized for patients with neurological symptoms or a high vascular-risk profile rather than performed indiscriminately, and in which incidental white matter changes are interpreted in the context of age and comorbidity rather than being automatically attributed to RA; such an approach may also foster closer multidisciplinary collaboration between rheumatologists, neurologists, and radiologists.

Several avenues warrant further investigation. Prospective, multicenter studies using standardized MRI protocols and validated visual rating scales—such as the Fazekas scale for white matter lesions—are needed to quantify the burden of CNS abnormalities and to disentangle the relative contributions of RA-specific inflammation and conventional vascular risk factors. Longitudinal designs that combine serial imaging with markers of disease activity, systemic inflammation, and endothelial function, and that incorporate detailed neurocognitive and neurological assessment, would clarify whether subclinical imaging findings translate into measurable clinical consequences over time [5,13]. The inclusion of age- and comorbidity-matched control groups, as well as RA patients managed with different treatment strategies, would help establish whether the patterns observed here are specific to RTX-treated disease or generalize across the RA population. Finally, the integration of quantitative and advanced neuroimaging techniques with candidate biomarkers of neuronal and glial injury may enable earlier and more specific identification of clinically relevant CNS involvement.

This study has several important limitations. First, the retrospective, single-center design and the modest sample of 84 patients limit statistical power and generalizability. Second, and most importantly, the study lacked both a healthy control group and a comparator group of RA patients not treated with RTX; consequently, we cannot determine whether the observed 71.5% prevalence of MRI abnormalities exceeds that expected in an age- and vascular-risk-matched population, nor can we attribute the findings specifically to RA or to RTX. Third, because all patients received RTX—typically reserved for more severe, long-standing, or treatment-refractory disease—the cohort is subject to selection bias that further limits generalizability. Fourth, although white matter hyperintensities were graded semiquantitatively using a Fazekas-type scale, fully quantitative lesion volumetry and automated segmentation were not performed, so lesion volume and total burden were not measured, and progression was assessed only qualitatively. Fifth, comorbidity was analyzed primarily as a composite binary variable, and several potentially relevant vascular confounders—including smoking, body mass index, blood-pressure control, lipid profile, and concomitant medication exposure—were not systematically available for adjustment; the limited number of outcome events also precluded more extensive multivariable modeling. Sixth, follow-up imaging was available in fewer than half of the patients, with non-standardized timing and indications, so the observation of temporal stability cannot be generalized to the whole cohort. Finally, standardized neurological and neurocognitive assessments were not performed, so the clinical significance of the observed MRI abnormalities—the central question motivating this study—could not be directly established. These limitations should be addressed in adequately powered, prospective, controlled studies. Conversely, because cranial MRI was obtained systematically for mandatory pre-treatment neurological clearance rather than for neurological symptoms, selection bias toward symptomatic patients was reduced, even though the eligibility requirement still restricted the cohort to rituximab-treated patients.

## 5. Conclusions

Cranial MRI abnormalities are frequently observed in RA patients treated with rituximab, predominantly as white matter changes, and were independently associated with older age and comorbidity burden rather than with RA-specific disease characteristics. In the absence of a control group, however, we cannot determine whether their prevalence exceeds that of a comparable non-RA population, and the findings should be interpreted as associations rather than causal relationships. Imaging findings remained stable in the subgroup that underwent repeat imaging, and no treatment-related demyelinating disease or PML was observed; this represents the absence of an observed imaging safety signal rather than evidence of the neurological safety of rituximab, since the study was not designed for that purpose. These findings emphasize the importance of cautious, contextual interpretation of cranial neuroimaging in RA. Adequately powered, prospective, controlled studies incorporating standardized quantitative imaging and neurocognitive assessment are needed to clarify the clinical significance of CNS abnormalities in RA.

## Figures and Tables

**Figure 1:**
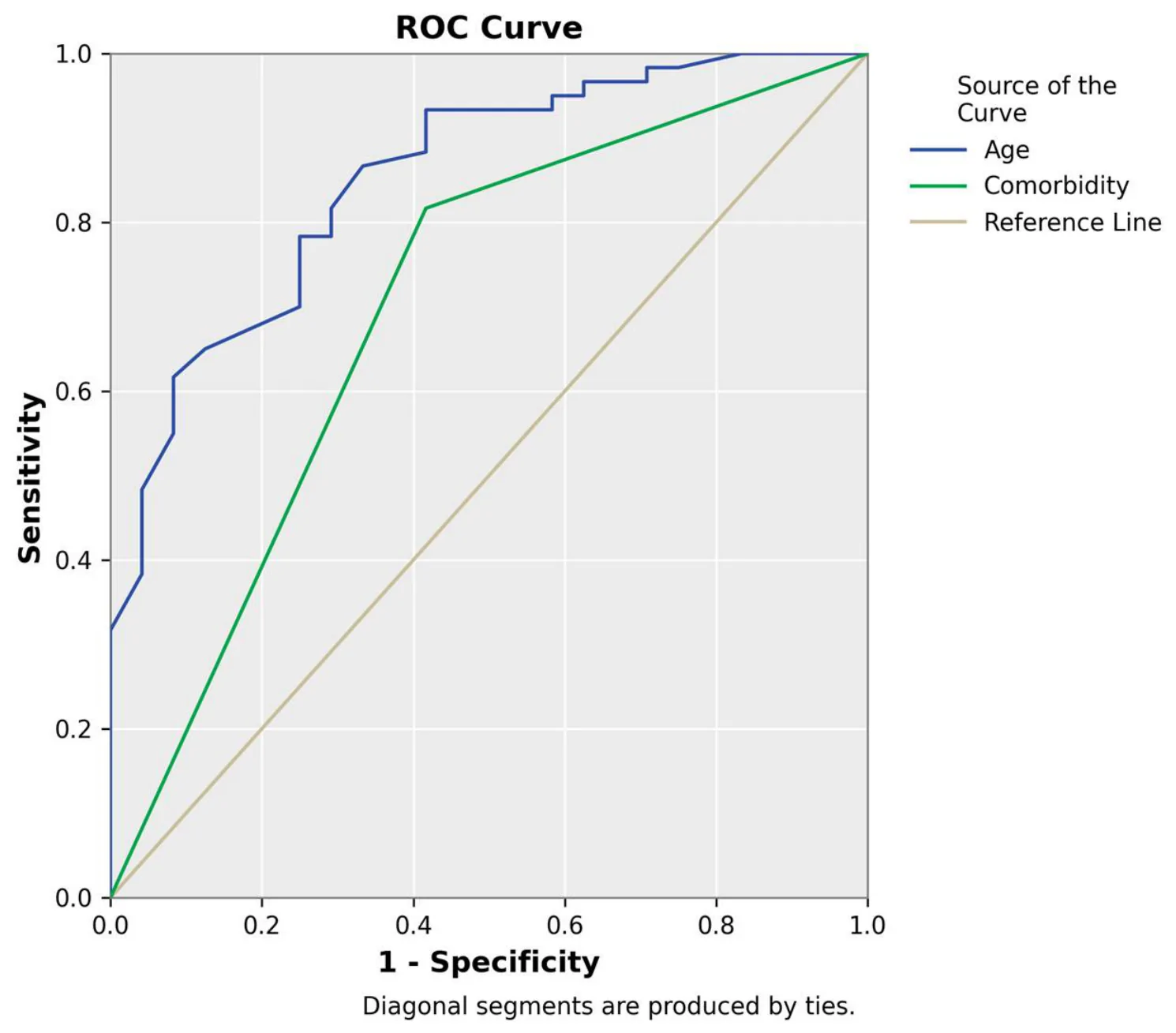
Receiver operating characteristic (ROC) curve analysis for the prediction of abnormal cranial MRI findings by age and comorbidities.

**Table 1:** Demographic and clinical characteristics of the study population (*n* = 84).

Variable	Value (*n* = 84)
Age (years), mean ± SD	54.69 ± 11.67
Sex, *n* (%)	
Female	73 (86.9)
Male	11 (13.1)
Disease duration (months), median (min–max)	154 (1–354)
RF positivity, *n* (%)	74 (88.1)
Anti-CCP positivity, *n* (%)	71 (84.5)
Comorbidity, *n* (%)	59 (70.2)
Hypertension	30 (35.7)
Diabetes mellitus	14 (16.7)
Hyperlipidemia	8 (9.5)
Coronary artery disease	6 (7.1)
Cerebrovascular disease	3 (3.6)
Chronic renal failure	1 (1.1)

Data are presented as mean ± standard deviation, median (minimum–maximum), or number (percentage), as appropriate. RF, rheumatoid factor; anti-CCP, anti-cyclic citrullinated peptide; SD, standard deviation.

**Table 2:** Comparison of patients according to cranial MRI findings (normal vs. abnormal) and to the presence of white matter disease (WMD).

Variable	MRI Normal (*n* = 24)	MRI Abnormal (*n* = 60)	*p*-Value	WMD Present (*n* = 48)	WMD Absent (*n* = 36)	*p*-Value
Age (years)	44.37 ± 10.04	58.81 ± 9.58	**<0.001**	58.72 ± 9.64	49.30 ± 12.09	**0.001**
Disease duration (months)	126 (18–354)	156.5 (1–314)	0.488	155 (1–312)	139.5 (16–354)	0.769
Sex, female, *n* (%)	21 (87.5)	52 (86.7)	0.846	43 (89.6)	30 (83.3)	0.517
Comorbidity, *n* (%)	10 (41.7)	49 (81.7)	**0.001**	39 (81.2)	20 (55.6)	**0.016**
RF positivity, *n* (%)	19 (79.2)	55 (91.7)	0.155	43 (89.6)	31 (86.1)	0.738
Anti-CCP positivity, *n* (%)	19 (79.2)	52 (86.7)	0.193	42 (87.5)	29 (80.6)	0.544
Extra-articular involvement, *n* (%)	12 (50.0)	26 (43.3)	0.813	20 (41.7)	18 (50.0)	0.510

Bold *p*-values denote statistical significance (*p* < 0.05). Continuous variables are shown as mean ± standard deviation or median (minimum–maximum); categorical variables are shown as number (percentage) within each group. WMD, white matter disease; RF, rheumatoid factor; anti-CCP, anti-cyclic citrullinated peptide.

**Table 3:** Semiquantitative (Fazekas-type) grading of white matter hyperintensities in the study cohort (*n* = 84).

White Matter Hyperintensity	Grade 1, *n* (%)	Grade 2, *n* (%)	Grade 3, *n* (%)	Any Grade, *n* (%)
Periventricular	4 (4.8)	4 (4.8)	0 (0.0)	8 (9.5)
Deep	28 (33.3)	12 (14.3)	1 (1.2)	41 (48.8)
Subcortical	7 (8.3)	5 (6.0)	0 (0.0)	12 (14.3)

White matter hyperintensities were graded with a Fazekas-type visual rating scale. Data are the number (percentage) of the 84 patients; because patients could have hyperintensities in more than one location, categories are not mutually exclusive.

**Table 4:** Multivariable logistic regression analysis of factors independently associated with abnormal cranial MRI findings.

Variable	Adjusted OR (95% CI)	*p*-Value
Age (per 1-year increment)	1.16 (1.08–1.25)	<0.001
Age (per 10-year increment)	4.49 (2.13–9.49)	<0.001
Comorbidity (present vs. absent)	5.36 (1.49–19.23)	0.010

Adjusted odds ratios (OR) with 95% confidence intervals (CI) from a multivariable binary logistic regression model with abnormal cranial MRI as the dependent variable. Associations were essentially unchanged after additional adjustment for sex and disease duration. OR, odds ratio; CI, confidence interval.

## Data Availability

The data that support the findings of this study are available from the corresponding author upon reasonable request.
